# Genotypes and Phenotypes of *DMD* Small Mutations in Chinese Patients With Dystrophinopathies

**DOI:** 10.3389/fgene.2019.00114

**Published:** 2019-02-18

**Authors:** Liang Wang, Min Xu, Huan Li, Ruojie He, Jinfu Lin, Cheng Zhang, Yuling Zhu

**Affiliations:** ^1^Department of Neurology, National Key Clinical Department and Key Discipline of Neurology, The First Affiliated Hospital, Sun Yat-sen University, Guangzhou, China; ^2^Department of Dermatology, Sun Yat-sen Memorial Hospital, Sun Yat-sen University, Guangzhou, China; ^3^Department of Dermatology, The Second Affiliated Hospital of Guangzhou Medical University, Guangzhou, China

**Keywords:** dystrophinopathies, small mutation, mutation spectrum, genotype–phenotype correlation, exon skipping therapy

## Abstract

Dystrophinopathies are a group of neuromuscular disorders resulting from mutations in *DMD*, including Duchenne muscular dystrophy (DMD), intermediate muscular dystrophy (IMD), and Becker muscular dystrophy (BMD). Herein, we present the characteristics of small mutations in Chinese patients with dystrophinopathies, and explore genotype–phenotype correlations. In our cohort, 115 patients with small mutations (18.49% of all patients) were included and *DMD* mutations were detected by either Sanger (53.91%) or next generation sequencing (46.09%). In total, 106 small mutations were detected, 28 of which (26.42%) had not been reported previously. The most common mutations were nonsense mutations (52.17%), followed by splicing (24.35%), frameshift (17.39%), and missense mutations (5.22%), in addition to a single untranslated region mutation (0.87%). We discovered distinct mutation characteristics in our patients, such as different positional distributions, indicating different exon skipping therapy strategies for small mutations in Chinese patients. Almost all patients (96.51%) with truncating or missense mutations, were covered by triple/double/single-exon skipping therapy; the most frequent single-exon skipping strategy was skipping exon 32, applicable for 8.51% of patients. Furthermore, splicing classification grades were correlated with phenotypes in nonsense mutations (*P* < 0.001), and serum creatinine levels differed significantly between DMD/IMD and BMD for patients ≤ 16 years old (*P* = 0.002). These observations can further aid prognostic judgment and guide treatment. In conclusion, the mutation characteristics and genotype–phenotype correlations in Chinese patients with dystrophinopathies and small mutations could provide insights into the molecular mechanisms of pathogenesis, diagnosis, and treatment designs.

## Introduction

Dystrophinopathies are X chromosome-linked recessive neuromuscular disorders with a pooled prevalence of 6.31 per 100,000 males (Mah et al., 2014), and are characterized by progressive muscle weakness, elevated levels of serum creatine kinase (CK), and death as a result of cardiorespiratory failure (Flanigan, 2014). These disorders can be classified as Duchenne muscular dystrophy (DMD; #310200), Becker muscular dystrophy (BMD; #300376), and intermediate muscular dystrophy (IMD). DMD has a heavy disease course with loss of ambulation before 13 years of age, whereas BMD exhibits mild progression and better prognosis (Bushby et al., 2010); the severity of IMD lies between that of DMD and BMD (Flanigan, 2014).

Dystrophinopathies result from mutations in *DMD* (OMIM: ^∗^300377). *DMD* encodes dystrophin, a structural protein with four domains: actin-binding domain (ABD), rod domain (RD), cysteine-rich domain (CRD), and C-terminal domain (CTD). Dystrophin is located in the myofiber membrane where it assists in membrane stability (Le Rumeur et al., 2010). The most common mutation type in dystrophinopathies involves large rearrangements, which account for 77.7% of mutations (Tuffery-Giraud et al., 2009). The remainder are *DMD* small mutations, mainly consisting of point mutations, small deletions, and small insertions (Tuffery-Giraud et al., 2009). Uncovering the mutation spectrum observed in specific diseases can lead to the development of genetic medicine, such as exon skipping therapy and nonsense read through therapy (Jarmin et al., 2014). The ‘reading-frame rule’ can explain approximately 91% of the genotype–phenotype correlations observed in patients with large rearrangements in *DMD* (Aartsma-Rus et al., 2006). However, the genotype–phenotype correlations of small mutations are more complicated (Aartsma-Rus et al., 2006); for example, nonsense mutations introduce premature stop codons (PTCs), which may result in truncated proteins and correlate with severe phenotypes (Ginjaar et al., 2000; Richards et al., 2015). However, an increasing number of recent studies have reported BMD cases with nonsense mutations (Ginjaar et al., 2000; Aartsma-Rus et al., 2006; Flanigan et al., 2011). Therefore, genotype–phenotype correlation with respect to *DMD* small mutations warrants further research.

Several studies have reported the mutation characteristics of small mutations but not focused on genotype–phenotype correlations in Chinese patients with dystrophinopathies (Chen et al., 2013; Yang et al., 2013; Guo et al., 2015). Therefore, we studied the characteristics of *DMD* small mutations and assessed genotype–phenotype correlations. Additionally, we examined the correlation between serum creatinine (SCRN) and phenotypes of patients with small mutations, as we had previously verified the correlation between SCRN and phenotypes of patients whose main mutations were large rearrangements (Zhang et al., 2015; Wang et al., 2017).

## Materials and Methods

### Participants

A total of 622 male patients with dystrophinopathies were diagnosed through clinical manifestations, biochemical changes, and genetic examinations between September 2012 and February 2018 at the First Affiliated Hospital, Sun Yat-sen University, China. Of these, 115 unrelated male patients with small mutations were included in this study. All of included patients were East Asian, with 104 of Han ethnicity, 1 Hui, and 10 of unknown ethnicities. Some subjects had also participated in our previous studies (Zhang et al., 2015; Wang et al., 2017).

### Phenotype Classification

Four phenotypic subgroups were defined as follows: DMD, IMD, BMD, and pending. These classifications were mainly based on clinical severity, such as age at which the patient lost ambulation (DMD < 13 years; BMD ≥ 16 years; and 13 years ≤ IMD < 16 years; Tuffery-Giraud et al., 2009). Some patients could not be classified in this manner, and thus those with an onset of weakness by the age of 5 were recognized as DMD, while those that retained nearly normal motor function, or had very mild motor dysfunction after the age of 5 were classified as BMD (Brooke et al., 1981; Marden et al., 2005).

Patients whose motor dysfunction severity fell between DMD and BMD were classified as IMD. This study included seven patients with IMD; two of whom lost ambulation at the age of 14 and another two (12 and 13 years old) had severe difficulty walking, manifested obvious waddling gaits, and lost the ability of standing up from seated positions. The remaining three patients were 11–12 years old and presented with obvious waddling gait as well as difficulties climbing stairs and, getting up off the ground or from a chair. They were classified as IMD in this study because their motor dysfunctions were milder than those with DMD and more severe than BMD patients at the same age. We could not completely exclude the possibility that the three IMD patients between 11–12 years old would lose ambulation before the age of 13, which would change their classifications to DMD. However, this does not affect our analysis because DMD and IMD were grouped as DMD/IMD during this study.

Patients whose phenotypes could not be determined or with missing disease history information (only one patient had missing clinical data) were classified as ‘pending.’

### SCRN and Genetic Examination

Blood collection was performed as previously described (Wang et al., 2017). Sera were immediately tested for CK and SCRN using enzymatic reactions in a Beckman Coulter AU5800 clinical chemistry analyser (Beckman Coulter, Brea, CA, United States). Generally, results of CK and SCRN levels obtained from the first test were included for analysis of patients with several follow-up serum tests. For *DMD* mutation analysis, blood (2 mL) was collected and then multiplex ligation-dependent probe amplification (MLPA) was used to detect large sequence rearrangements. Blood samples with negative MLPA results were then checked for small-scale mutations by next-generation sequencing (NGS) using an Illumina HiSeq 2000 platform (Illumina, San Diego, CA, United States) or Sanger sequencing as previously described (Chen et al., 2013; Wang et al., 2017). Sanger sequencing was used to detect whether the probands’ mothers carried the *DMD* mutation. The *DMD* transcript number is NM_004006.2.

### Muscle Histology

Twenty-five patients underwent muscle biopsy and then the muscle samples were used for immunohistochemistry. The quadriceps femoris and gastrocnemius muscles were extracted for analysis as previously (Yu et al., 2017). The antibody against dystrophin was purchased from Novocastra (Newcastle, United Kingdom). We analyzed expression levels of the dystrophin C-terminus as using antibodies against this C-terminus is a highly reliable diagnostic approach; furthermore, the dystrophin C-terminus is translated in patients with nonsense mutations if beneficial spontaneous exon skipping occurred (Doriguzzi et al., 1997; Flanigan et al., 2011).

### Genetic Analysis

The Leiden Open Variation Database (LOVD), Universal Mutation Database (UMD)-DMD Database, ClinVar database, and Google were used to detect whether mutations had been previously reported. The pathogenicity of a mutation was evaluated according to the guidelines of the American College of Medical Genetics and Genomics (Richards et al., 2015); the pathogenic or likely pathogenic mutations were included in this study. Domains where mutations were located were defined as ABD (exons 1–8), RD (exons 9–62), CRD (exons 63–69), and CTD (exons 70–79; Engel, 2004). The Leiden DMD reading frame checker^1^ was used to determine whether skipping the exon in which the mutation was located would lead to an open reading frame (ORF) shift [ORF shift (out-of-frame) was set as 0, otherwise it was 1 (in-frame)] (Aartsma-Rus et al., 2006). Mechanisms explaining spontaneous exon skipping of nonsense mutations varied; however, a splicing change resulting from the disruption or creation of a splicing regulatory element (SRE) appeared to be the primary mechanism that mainly occurred via exonic splicing silencer (ESS) and exonic splicing enhancer (ESE) elements (Flanigan et al., 2011). Human Splicing Finder (HSF^2^) was used to determine changes in ESS and ESE elements in mutated *DMD* (Desmet et al., 2009). The HSF score was 0 when there were no SRE changes and 1 when a new ESS or broken ESE was present. Splicing classification grades were obtained by multiplying ORF changes by the HSF score as beneficial exon skipping is based on in-frame changes after exon skipping. Grade 0 represents an absence of exon skipping or unbeneficial exon skipping that leads to changes in the reading frame, both of which produce PTCs; grade 1 represents beneficial exon skipping.

### Statistical Analysis

Data were analyzed using SPSS 24.0 (IBM, Armonk, NY, United States) and GraphPad Prism 6 (GraphPad Software, San Diego, CA, United States) software. Microsoft Excel 2016 (Microsoft, Redmond, Washington, DC, United States) was also used for plotting information. Contingency tables and Chi square tests were used for categorical variables. When any expected value of contingency tables was less than 5, the results were corrected for continuity, when the value was less than 1 or the total sample number was less than 40, the Fisher exact test was used. For statistical significance, odds ratios (ORs) were reported with 95% confidence intervals (CIs) in 2 × 2 contingency tables. Correlation between phenotypes and factors (age of examination, mutation type and SCRN, or CK levels) were analyzed using logistic regression. *P* < 0.05 was considered statistically significant.

## Results

### Phenotypes and Mutation Characteristics

In our cohort of patients with dystrophinopathies (622), 115 (18.49%) presented with small mutations. Among them, DMD was observed in 59 subjects (51.30%), BMD in 31 (26.96%), and IMD in 7 (6.09%), while 18 were classified as pending (15.65%). We categorized DMD and IMD patients into one DMD/IMD group due to prognostic similarities and the low number of IMD.

**FIGURE 1:**
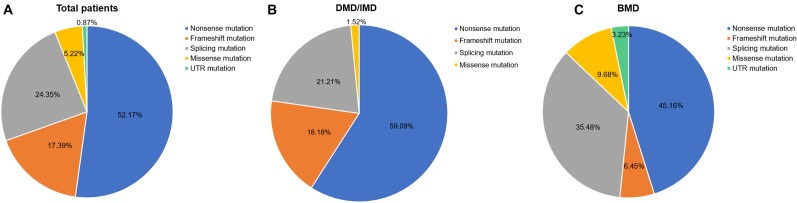
Distribution of mutation types. The ratios of mutation types in **(A)** all patients, **(B)** DMD/IMD, and **(C)** BMD.

**FIGURE 2:**
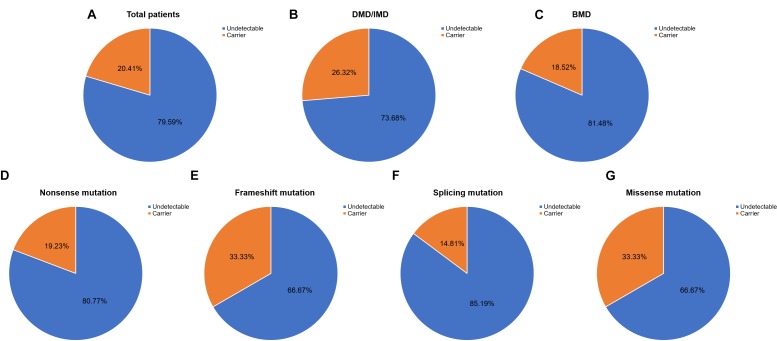
Carrier status of probands’ mothers. Carrier status ratios for mothers of **(A)** all probands, **(B)** DMD/IMD, and **(C)** BMD as well as probands with the following different mutation types, **(D)** nonsense, **(E)** frameshift, **(F)** splicing, and **(G)** missense mutations.

Out of 115 patients, 106 mutations were detected, 28 of which (26.42%) had not been reported previously (search date: 2018/5/2). Small mutations in 62 (53.91%) and 53 (46.09%) subjects were detected by Sanger sequencing and NGS, respectively. There were seven common mutations; furthermore, the phenotypes were not noticeably different between patients with the same mutations (Table 1 and see Supplementary Table S1 for detailed patient information). As our study patients were unrelated, we analyzed genotype and phenotype characteristics based on the number of patients. The most common types of mutations were nonsense mutations (60/115, 52.17%), followed by splicing (28/115, 24.35%), frameshift (20/115, 17.39%), and missense mutations (6/115, 5.22%). A single untranslated region (UTR) mutation (1/115, 0.87%) was also detected. Of all patients with truncating mutations, those with nonsense mutations (60/80, 75.00%) were more common than those with frameshift mutations (20/80, 25.00%). Differences in mutation types were observed between DMD/IMD and BMD (*P* = 0.032; Figure 1). Additionally, carrier status of the probands’ mothers was determined for 78 out of 98 females (79.59%) whose DNA was submitted for Sanger sequencing; carrier status was not significantly different between DMD/IMD and BMD (*P* = 0.433) or patients with different mutations (*P* = 0.543; Figure 2).

**Table 1:** Phenotypes of patients with the same mutations.

Mutation		DMD	IMD	BMD	Pending
Nonsense mutation	c.6292C > T	2	0	0	0
	c.5632C > T	0	0	2	0
	c.5287C > T	0	0	2	0
	c.10141C > T	3	1	0	0
Splicing mutation	c.5740-1G > T	0	0	2	0
	c.2804-1G > T	1	1	0	0
	c.186+2T > C	0	0	1	1

*DMD, Duchenne muscular dystrophy; IMD, intermediate muscular dystrophy; BMD, Becker muscular dystrophy.*

**FIGURE 3:**
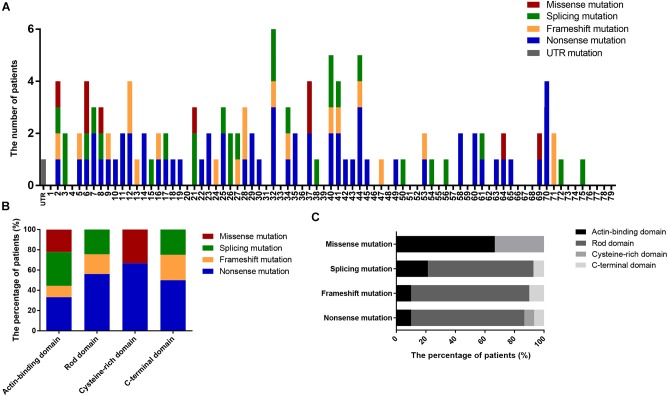
Positional distributions of small mutations. **(A)** Distribution of exons in which mutations are located. **(B)** The ratios of mutation types in *DMD* domains. **(C)** Distribution of domains in which mutations are located. Distributions of splicing mutation were classified to consensus exons.

We also determined the distribution of mutations in *DMD* (Figure 3A) and found that nearly 50% of patients had mutations located in the 5′ region upstream of exon 30 (4071 bp; 36.82% of the gene) in the *DMD* coding sequence. Additionally, the distribution of mutation types in *DMD* domains differed significantly (*P* = 0.002; Figure 3B,C); no splicing and frameshift mutations were observed in the CRD, and no missense mutations were observed in the RD or CTD.

### Nonsense Mutations

**FIGURE 4:**
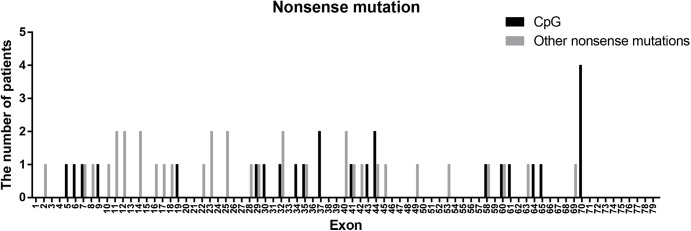
Positional distribution of nonsense mutations located in CpG dinucleotides and other regions.

Nonsense mutations (60/115) were the most common across all mutation types (52.17%) and when truncating mutations were isolated (60/80, 75.00%). Patients with transition mutations (41/60, 68.33%) were more common than those with transversion mutations (19/60, 31.67%). C-to-T (37/41, 90.24%) and G-to-A (4/41, 9.76%) were the only transition types observed. Similar to the results of a previous study (Tuffery-Giraud et al., 2009), the frequency of patients with mutations located in CpG dinucleotides was 41.67% (25/60; consisting of 23 C-to-T, 1 C-to-A, and 1 G-to-A). These results indicate that CpG islands are a mutational hot spot, likely due to oxidative deamination of 5-methyl cytosine (Tuffery-Giraud et al., 2009). However, the distribution of CpG mutations observed in this study differed from what was previously reported (Figure 4). The most common CpG mutation we observed was c. 10141C > T (4/25, 16.00%) in exon 70, followed by c. 5287 C > T (2/25, 8.00%) in exon 37, and c. 6292 C > T (2/25, 8.00%) in exon 44. Furthermore, we observed mutations at CpG dinucleotides located in exon 9 (c.883C > T), exon 32 (c.4375C > T), and exon 61 (c.9100C > T), which were not reported in a previous study (Tuffery-Giraud et al., 2009).

Nonsense mutation sites were primarily clustered at the first codon position (49/60, 81.67%), followed by the third (7/60, 11.67%), and second position (4/60, 6.66%). No G-to-T substitutions at the first codon position were located at the CpG dinucleotide; however, C-to-T at the first codon position were more often observed at CpG dinucleotides than at any other site [23 mutations (62.16%) vs. 14 mutations (37.84%), respectively]. Analysis of PTC types observed in nonsense mutations revealed that TGA was the most common (29/60, 48.33%), followed by TAG (15/60, 25.00%), and TAA (16/60, 26.67%). Moreover, there was a statistically significant difference between these PTC types among the different substitutions observed at the first codon position (*P* < 0.001; Table 2).

**Table 2:** Base substitutions observed in nonsense mutations.

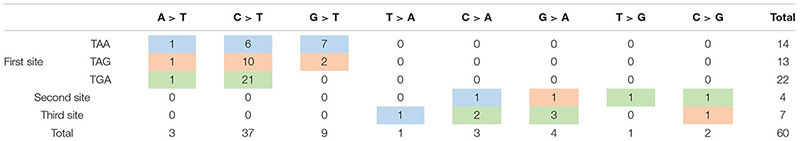

*Substitutions in blue boxes lead to TAA codon; substitutions in yellow boxes lead to TAG codon; and substitutions in green boxes lead to TGA codon*.

### Frameshift Mutations

**FIGURE 5:**
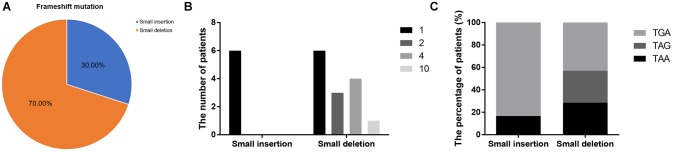
Information regarding small insertions and deletions in frameshift mutations. **(A)** Proportions of small insertions and deletions in frameshift mutations. **(B)** The number of deleted or inserted bases in small insertions and deletions. **(C)** Proportions of first hidden premature termination codons in small insertions and deletions.

**FIGURE 6:**
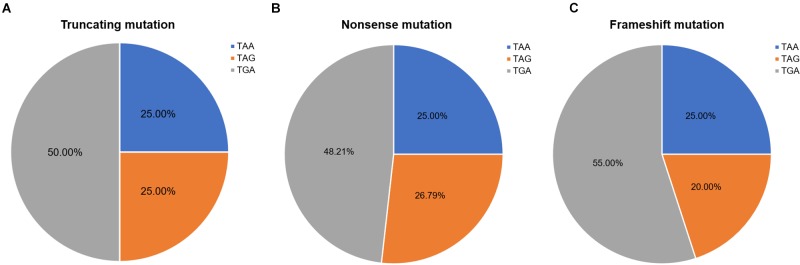
Ratios of premature termination codons. The constituent ratios of premature termination codons in **(A)** truncating, **(B)** nonsense, and **(C)** frameshift mutations.

Frameshift mutations accounted for 25.00% of all truncating mutations. Within this group, there were 14 small deletions (70.00%) and 6 small insertions (30.00%; Figure 5A). Twelve mutations (60.00%) resulted from either small deletions or insertions of a single base; however, no small insertions of several bases were observed (Figure 5B). Apart from a small insertion (c.1376dupA) and deletion (c.1533_1536del) in exon 12, no exon contained both a small insertion and deletion. The mRNA of transcripts containing frameshift mutations were degraded by nonsense-mediated decay due to the presence of a hidden downstream PTC (Miller and Pearce, 2014). Analysis of the first hidden PTC downstream of the mutated sites identified 11 TGA (55.00%), 5 TAA (25.00%), and 4 TAG (20.00%) codons. These results did not differ from the PTC distribution results in the nonsense mutation group (*P* > 0.05; Figure 6). However, distribution of the first hidden PTC in small insertions and deletions differed but was not statistically significant (*P* = 0.361; Figure 5C). The positional distributions were significantly different between small insertions and deletions (*P* = 0.003); all small deletions were located in the RD, while two small insertions were observed in each of the ABD, RD, and CTD.

### Splicing Mutations

**FIGURE 7:**
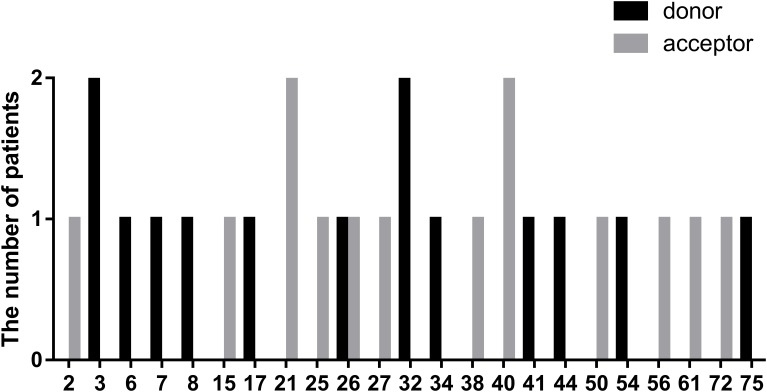
Positional distribution of splicing mutations located in donor and acceptor sites.

In this study, we defined splicing mutations as those within introns that led to *DMD* splicing changes. Out of 28 patients with splicing mutations, 21 mutations (75.00%) were located at a highly conserved splice site (±1–2 bp from the exon-intron boundary), whereas, the others (5/28, 17.86%) were located 3–5 bp from this boundary. In addition, we identified two splicing mutations, c.9164-3_9164-1delCAG and c.10329-11_10330del, that spanned highly conserved splice regions. Of all splicing mutations, substitutions were the most common mutation type observed (24/28, 85.72%), followed by small insertions (2/28, 7.14%), and deletions (2/28, 7.14%). Furthermore, there were 14 splicing mutations each (50.00%) in the splice acceptor and donor sites. The distribution of splicing mutations is represented in Figure 7.

### Missense Mutations

There were six missense mutations (5.22%) identified in this study, which is consistent with the percentages reported in previous studies (1–5%; Sedlácková et al., 2009; Tuffery-Giraud et al., 2009; Juan-Mateu et al., 2015). Four of these mutations were located in the ABD and the other two in the CRD, consistent with the clustering phenomenon of missense mutation in ABD and CRD demonstrated in previous reports (Tuffery-Giraud et al., 2009; Juan-Mateu et al., 2015). Although the RD accounts for 75.92% of *DMD*, missense mutations were rare in this domain. Compared with the rate of missense mutations, the proportions of other mutations in the RD correlate with its length (nonsense mutations, 76.67%; frameshift mutations, 80.00%; and splicing mutations, 71.43%; Figure 3C). This phenomenon may be associated with the spectrin-like repeat structure in RD.

### Dystrophin Expression in the Muscles

Twenty-five patients underwent muscle biopsy; eight presented with patchy dystrophin staining in muscles, whereas the others showed absence of dystrophin expression. Patchy staining represents dystrophin expression levels > 10%. Among the 8 patients with patchy staining, 7 manifested BMD and 1 was pending. For patients with absence of dystrophin expression, 15 manifested DMD/IMD, 1 BMD, and 1 was pending (see Supplementary Table S1 for detailed information).

### Levels of SCRN Differ in Patients With DMD/IMD and BMD

**FIGURE 8:**
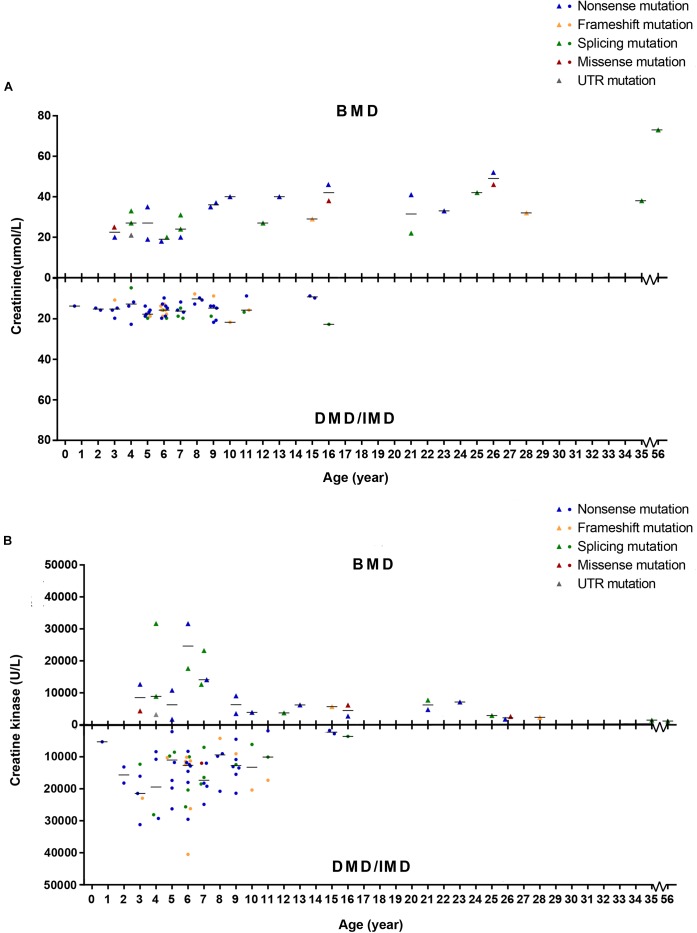
Correlations between phenotypes and serum creatinine (SCRN) or creatine kinase (CK) levels. **(A)** SCRN levels in patients with Duchenne muscular dystrophy (DMD)/intermediate muscular dystrophy (IMD) or Becker muscular dystrophy (BMD). **(B)** CK levels in patients with DMD/IMD or BMD. The median of SCRN levels at each age is represented by short lines.

To detect whether SCRN and CK levels differed between phenotypes, we set phenotypes as dependent variables while age of examination, mutation type and SCRN, or CK were independent variables and analyzed via logistic regression. Because all patients above the age of 16 presented with BMD, only ≤16 years old (*n* = 86) were included in this analysis. Furthermore, 10 subjects (11.63%) with missing SCRN data were excluded from this analysis. The results indicated that SCRN (*P* = 0.002, OR = 1.934, CI: 1.266–2.956) levels differed between DMD/IMD and BMD, whereas CK levels did not (*P* > 0.05). Moreover, SCRN levels did not differ between mutation types (*P* > 0.05; Figure 8).

### Genotype–Phenotype Correlations

Dystrophinopathy phenotypes are dependent on the degree to which the dystrophin gene, *DMD*, is affected by the mutation. Complete loss of *DMD* function leads to DMD, while relatively mild disruption of *DMD* function leads to BMD (Monaco et al., 1988). Our results indicate that fewer DMD/IMD patients had non-truncating mutations (15/66, 22.73%) compared with those with BMD (15/31, 48.39%; *P* = 0.011, OR = 0.314, CI: 0.126–0.779). These results support the theory that mutations with a greater effect on protein function result in more severe phenotypes because truncating mutations usually lead to degeneration of mRNA and complete absence of the protein (Miller and Pearce, 2014).

We further analyzed the correlation between phenotypes and the protein domains in which the mutations were located (Table 3). We found no correlation between phenotypes and domains in which mutations are located for splicing and missense mutations (*P* > 0.05). However, all the patients with frameshift mutations in RD presented with DMD/IMD, which was significant (*P* = 0.033).

**Table 3:** Domain distribution of mutations in DMD/IMD and BMD.

	UTR	Nonsense mutation				Frameshift mutation				Splicing mutation				Missense mutation			
		ABD	RD	CRD	CTD	ABD	RD	CRD	CTD	ABD	RD	CRD	CTD	ABD	RD	CRD	CTD
DMD/IMD	0	4	27	4	4	1	11	0	0	2	11	0	1	1	0	0	0
BMD	1	0	14	0	0	1	0	0	1	2	9	0	0	2	0	1	0

*DMD, Duchenne muscular dystrophy; IMD, intermediate muscular dystrophy; BMD, Becker muscular dystrophy; UTR, untranslated region; ABD, actin-binding domain; RD, rod domain; CRD, cysteine-rich domain; CTD, C-terminal domain.*

**FIGURE 9:**
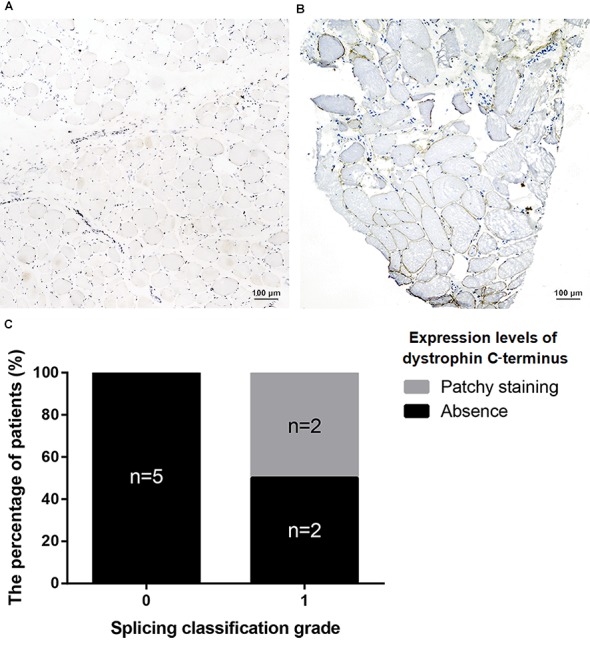
Correlation between splicing classification grades and dystrophin levels in patients with nonsense mutations. Expression levels of dystrophin C-terminus were evaluated using muscle immunohistochemistry with antibody against this terminus. Two staining conditions were observed in the muscle sections: **(A)** absence staining and, **(B)** patchy staining. **(C)** Bar chart representing percentage of patients versus splicing classification grade.

All nonsense mutations identified in BMD were located in the RD (Table 3). Given the high proportion of exons whose deletions lead to in-frame mutations (30/54, 55.56%) and the probable mild phenotypes resulting from in-frame deletions at RD (Yokota et al., 2012), spontaneous exon skipping may account for BMD with nonsense mutations. To assess the applicability of splicing classification grades for predicting spontaneous exon skipping in nonsense mutations, we analyzed the correlation between splicing classification grades and expression levels of the dystrophin C-terminus. All nonsense mutations with splicing classification grade 0 exhibited absent dystrophin levels, whereas half of nonsense mutations with splicing classification grade 1 presented with patchy dystrophin C-terminus staining (Figure 9). Thus, our results suggest that nonsense mutations with splicing classification grade 0 did not lead to beneficial spontaneous exon skipping.

Furthermore, we found that nonsense mutations with splicing classification grade 0 most frequently represented DMD/IMD (26/28, 92.86%, *P* < 0.001, OR = 12.000, CI: 2.332–61.758). All nonsense mutations with splicing classification grade 0 at the ABD, CRD, and CTD, were associated with DMD/IMD (Table 4). Analysis of nonsense mutations at the RD revealed that those with splicing classification grade 0 also most frequently represented DMD/IMD (15/17, 88.24%, *P* = 0.011, OR = 7.500, CI: 1.400–40.178). Thus, splicing classification grades were helpful in distinguishing DMD/IMD from BMD with nonsense mutations.

**Table 4:** Correlation between splicing classification grade and phenotypes in nonsense mutation cases.

	ABD		RD		CRD		CTD	
Splicing classification grade	0	1	0	1	0	1	0	1
DMD/IMD	3	1	15	12	4	0	4	0
BMD	0	0	2	12	0	0	0	0

*DMD, Duchenne muscular dystrophy; IMD, intermediate muscular dystrophy; BMD, Becker muscular dystrophy; UTR, untranslated region; ABD, actin-binding domain; RD, rod domain; CRD, cysteine-rich domain; CTD, C-terminal domain.*

### Exon Skipping Strategy for Patients With Dystrophinopathies and Small Mutations

Exon skipping is a promising treatment for dystrophinopathies and can target small mutations by skipping the exons in which the mutations are located (Aartsma-Rus et al., 2009). If skipping a single exon containing the mutation resulted in a frameshift, adjacent exons can also be skipped to maintain the ORF (Yokota et al., 2012). Previous studies confirmed that exon skipping therapy can restore dystrophin in dystrophic mice and in muscle cells of patients with *DMD* small mutations (Aartsma-Rus et al., 2003; Fletcher et al., 2007; Spitali et al., 2009). In this study, we included truncating (nonsense and frameshift mutations) and missense mutations for our analysis of exon skipping therapy. The rationale for this is that splicing mutations can lead to spontaneous exon skipping or intron retention, which makes exon skipping strategies unreliable depending on the specific DNA changes (Thi Tran et al., 2005; Hagenbuch et al., 2010).

**FIGURE 10:**
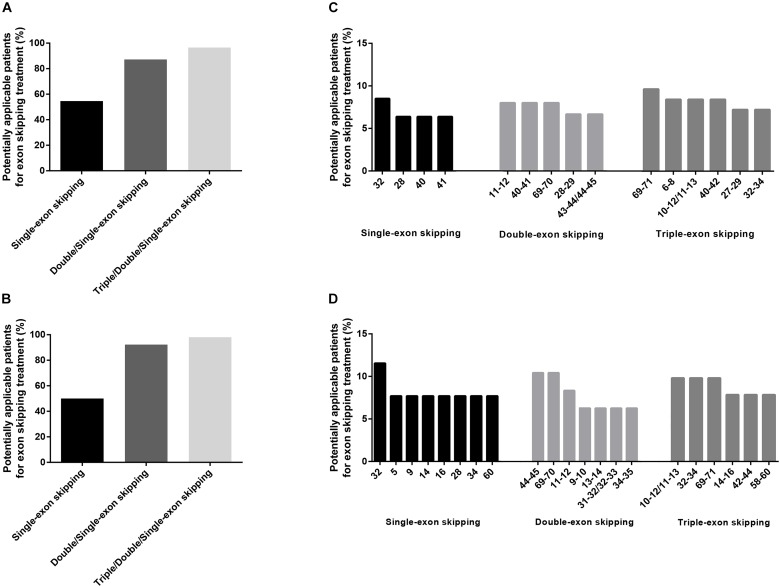
Exon skipping strategies for truncating and missense mutations. The covering proportions of potentially applicable patients among **(A)** all patients, and **(B)** those with Duchenne muscular dystrophy (DMD)/intermediate muscular dystrophy (IMD). Exon skipping strategies for **(C)** all patients and **(D)** those with DMD/IMD.

We found that single-exon skipping, single or double-exon (double/single-exon) skipping, and single, double, or triple-exon (triple/double/single-exon) skipping can cover 54.65, 87.21, and 96.51% of patients with truncating and missense mutations, respectively (Figure 10A). The aim of exon skipping treatment is to change severe phenotypes to milder ones. Theoretically, as BMD already represents the mildest phenotype, patients with BMD should not receive this treatment (Aartsma-Rus et al., 2009). Thus, after excluding BMD and pending subjects from our analysis, we found that single-exon, double/single-exon, and triple/double/single-exon skipping can cover 50.00, 92.31, and 98.08% of DMD/IMD patients with truncating and missense mutations (*n* = 52), respectively (Figure 10B). Excluding mutations representing BMD resulted in different exon skipping strategies with more coverage than that observed when BMD was included (Figure 10C,D).

## Discussion

The *DMD* gene is the largest known human gene, containing 79 exons and is 2.4 Mb in length (Kole and Krieg, 2015). MLPA for detecting *DMD* large rearrangements is an already proven practical and reliable method (Aartsma-Rus et al., 2016). On the other hand, sanger sequencing for detecting small mutations is costly and time-consuming due to the *DMD* gene length, making muscle biopsy important for diagnosis of dystrophinopathies (Bushby et al., 2010). In recent years, however, the development of NGS made the detection of *DMD* small mutations more cost-effective and accurate than before. Furthermore, since obtaining information about mutations is critical for prenatal diagnosis, preimplantation genetic diagnosis and administration of gene therapies (Jarmin et al., 2014), genetic testing is now performed prior to muscle biopsy for diagnosing dystrophinopathies, as stated in the latest guideline (Birnkrant et al., 2018). Considering the expensive cost of directly combining MPLA and sequencing, we initially perform MLPA for large rearrangements, which covers about 80% of patients. If the results are negative, we perform sequencing, thereby cutting down on cost. After the confirmation of probands’ mutations, the probands’ mothers should be verified for the carrier state. As sequencing technology develops and becomes cheaper, large rearrangements and small mutations can be detected simultaneously in the future.

Although muscle biopsies for detecting dystrophin expression are not critical for the diagnosis of dystrophinopathies nowadays, quantification of dystrophin expression levels is helpful in classifying phenotypes. Duchenne patients have little to no dystrophin in muscle tissue, while dystrophin levels in IMD and BMD are higher (Hoffman et al., 1988; Fanin et al., 1992, 1995; Angelini et al., 1994). Furthermore, low levels of dystrophin expression in female carriers suggests a poor prognosis (Pegoraro et al., 1995). Thus, dystrophin expression levels are correlated with clinical phenotypes. Our results were compatible with this theory, with the exception of a BMD patient that lacked dystrophin expression. Previous studies that included patients and DMD dog models also reported some exceptions (Castro-Gago, 2015; Vieira et al., 2015). It is difficult to explain this phenomenon, but it is possible that a supplementary mechanism is responsible for the dystrophin defect, which should be investigated further and may be a novel target for treating patients with dystrophinopathies.

Creatinine is a metabolic product of the creatine pathway, which is important for myofiber energy metabolism (Wyss and Kaddurah-Daouk, 2000). We found that SCRN levels in BMD patients younger than 16 years were higher than those in DMD/IMD, which was independent of mutation types. Furthermore, the difference of SCRN levels between DMD/IMD and BMD showed an increasing trend in an age-dependent manner. Therefore, SCRN levels correlate with clinical phenotypes, rather than genotypes, confirming the results of our previous small sample study (Wang et al., 2017). CK is a typical biomarker in neuromuscular disorders and whose levels are extremely elevated in dystrophinopathies (Pernice et al., 1986). Although CK is a good biomarker for screening and diagnosis, analysis of its levels could not distinguish DMD/IMD from BMD, which may result from the large variability in CK levels.

Regarding dystrophinopathy mutations, the characteristics revealed in this study are consistent with those of previous studies, including proportion of mutation and PTC types, frequency of nonsense mutations in CpG dinucleotides, and the ratios of small deletions and insertions (Sedlácková et al., 2009; Tuffery-Giraud et al., 2009; Magri et al., 2011; Juan-Mateu et al., 2015). The most common mutation type was nonsense mutations, which suggests that nonsense read-through therapies hold promise in China. However, we identified some special characteristics in our cohort. For example, base changes in the first codon position were correlated with PTC types in nonsense mutations, and positional distributions differed between small deletions and insertions.

Furthermore, the positional distribution of small mutations observed in this study differed from those reported previously (Flanigan et al., 2015; Juan-Mateu et al., 2015). In general, small mutations tended to cluster in the 5′ region of the *DMD* ORF. Additionally, the most common positions for small mutations were exon/intron 32, 40, and 44, which were different from those reported in other studies (Sedlácková et al., 2009; Flanigan et al., 2015; Guo et al., 2015; Juan-Mateu et al., 2015; Li et al., 2015). This difference can be observed between different countries, such as China, Spain, America, and the Czechia (Sedlácková et al., 2009; Flanigan et al., 2015; Juan-Mateu et al., 2015). As for the positional distribution of nonsense mutation, the top three in our cohort were exon 70, 32, and 44, similarly, different characteristics of distribution can be observed between different countries (Sedlácková et al., 2009; Tuffery-Giraud et al., 2009; Flanigan et al., 2015; Guo et al., 2015; Juan-Mateu et al., 2015; Li et al., 2015). However, the proportion of patients with nonsense mutations in exon 70 was the highest in almost all studies, except for a Spanish cohort (Sedlácková et al., 2009; Tuffery-Giraud et al., 2009; Flanigan et al., 2015; Guo et al., 2015; Juan-Mateu et al., 2015; Li et al., 2015). Thus, as the distribution characteristics of small mutations are distinguished in different regions, different strategies should be tailored for targeted genetic treatment.

We examined the utility of exon skipping therapy as an example of such targeted treatment. Our results indicated that exon skipping therapy can be used in truncating and missense mutation cases because single-exon skipping can cover half of all patients with these mutations while triple/double/single-exon skipping can cover 96.51%; this is consistent with previous reports (Aartsma-Rus et al., 2009; Yokota et al., 2012). On the other hand, the most frequent single-exon skipping strategy was exon 32 skipping (covering 8.51% of total patients and 11.54% of DMD/IMD patients with truncating and missense mutations), which was different from a previous study that only focused on nonsense mutations (Yokota et al., 2012). In theory, multiexon skipping can cover many more patients than can single-exon skipping; however, its application is challenging owing to toxicity and non-specificity (Aartsma-Rus et al., 2014). Thus, if strategies were developed to eliminate the associated toxicity and increase specificity, a multi-exon skipping strategy would be the preferred choice in the treatment of small mutations. All mutations that could not be treated by triple/double/single-exon skipping were located in exon 2, which might require exon 2–19 skipping (Yokota et al., 2012).

Theoretically, patients with BMD should not receive exon skipping therapy because the treatment aim is to reduce the phenotype severity of DMD to that of BMD (Aartsma-Rus et al., 2009). Therefore, it is important to evaluate the specific phenotype of patients before initiating treatment. The classical method of phenotypic classification involves evaluating motor dysfunction and measuring dystrophin levels in the muscles (Arahata et al., 1989). However, these classical methods have some disadvantages, such as inaccuracies for young patients and invasiveness, which limit their application (Wang et al., 2017). Our results suggest two supplementary methods that can be used to more accurately determine dystrophinopathy phenotypes. First, we showed that patients with truncating mutations are more likely to present as DMD/IMD. Moreover, patients with nonsense mutations with a splicing classification grade 0 have an increased likelihood of presenting as DMD/IMD (92.86%); this is because nonsense mutations with beneficial spontaneous exon skipping can convert severe phenotypes to milder phenotypes (Flanigan et al., 2011). We believe these findings may promote the clinical application of bioinformatic tools. Second, SCRN levels can be helpful in determining phenotypic classifications (Wang et al., 2017), and remains practicable for small mutations.

In some circumstances, such as for very young patients, phenotypes cannot be determined precisely, which makes the exon skipping strategy of initially analyzing total patients with dystrophinopathies. However, once the patient can be classified into a specific phenotype with the help of clinical manifestations, biomarkers, bioinformatic analysis, and other methods, BMD patients should be excluded from exon skipping therapy. Thus, we analyzed exon skipping strategies in all patients as well as in DMD/IMD patients only. The results indicated that the covering rates of exon skipping strategies for DMD/IMD were similar to those calculated when taking into account all patients. However, the exon skipping strategies with the greatest covering frequencies differed when patients with BMD and pending were excluded from the analysis. These strategies are important and thus close attention is required when medications are designed. After comprehensive analysis, our results indicate that exon skipping strategies as treatment options for small mutations in Chinese patients should focus on exon 32 for single-exon skipping, exons 69–70 for double-exon skipping, and exons 69–71 for triple-exon skipping. All three strategies were the most common across all patients and patients with DMD/IMD.

Finally, there are some limitations to this study. First, deep regions in introns could not be detected due to the limitations of current sequencing technologies. Therefore, the frequency of splicing mutations may be underestimated. Second, although we performed a preliminary investigation of spontaneous exon skipping for nonsense mutations using immunohistochemistry staining of muscle dystrophin, the number of patients that underwent biopsies was limited due to its invasiveness; thus, this topic must be studied further with a larger sample size. Furthermore, the quantification of muscle dystrophin levels detected by western blotting is helpful for classifying phenotypes, which was not applied in this study due to the few number of available muscle samples. Third, the number of patients under the age of 3 subjected to the CK and SCRN test was limited because patients at these ages are pauci-symptomatic and doctors are rarely consulted during this time.

## Conclusion

In this study, we identified 106 small mutations in 115 unrelated patients with dystrophinopathies and reported the mutation characteristics and genotype–phenotype correlations. These characteristics suggested a mutation pattern in the *DMD* gene. Furthermore, compared with other non-splicing mutations, nonsense mutations tended to result in splicing changes, which provided evidence for spontaneous exon skipping in BMD patients with nonsense mutation. One BMD patient exhibited a lack of dystrophin expression, suggesting a novel mechanism for phenotype modification that should be investigated further.

The novel mutations uncovered here will be helpful in the diagnosis of dystrophinopathies and the use of splicing classification grades for predicting phenotypes, indicating a practical clinical application for bioinformatic tools. Moreover, the classification of phenotypes with the help of splicing classification grades and SCRN levels can aid the judgment of prognosis and in the design of treatment strategies. Data regarding mutation distribution and the investigation of exon skipping strategies may offer information for drug design, as 96–98% of patients with truncating or missense mutations were covered by triple/double/single-exon skipping, supporting the potential of exon skipping therapy for dystrophinopathy treatment.

Thus, our study findings have expanded our knowledge and understanding of mutation characteristics and genotype–phenotype correlations in dystrophinopathies, which can provide further insights regarding its pathogenesis, diagnosis, and treatment design.

## Ethics Statement

The study was conducted according to the ethical guidelines of the Declaration of Helsinki (1975), and a waiver of informed parental or personal consent was approved by the ICE for Clinical Research and Animal Trials of the First Affiliated Hospital of Sun Yat-sen University.

## Author Contributions

LW and MX designed the study, analyzed the data, and drafted the manuscript. LW, HL, YZ, RH, and JL recorded clinical and genetic data. YZ and CZ supervised the study and assisted in drafting and revising the manuscript.

## Conflict of Interest Statement

The authors declare that the research was conducted in the absence of any commercial or financial relationships that could be construed as a potential conflict of interest.

## Footnotes

1 http://www.dmd.nl/index.html

2 http://www.umd.be/HSF3/index.html
